# Transforming Emergency Medicine Mentorship: A Skill-Building Curriculum to Optimize Mentee Growth and Development

**DOI:** 10.5070/M5.523833

**Published:** 2026-10-31

**Authors:** Leah G Reznick, Nicole A Steckler

**Affiliations:** * Oregon Health & Science University, Portland, OR

## Abstract

**Audience and type of curriculum:**

This mentorship training curriculum is designed for faculty seeking to improve mentoring skills in an academic health system setting. The curriculum offers interactive small-group skill practice supported by brief didactic lectures, paired peer mentoring, and large group reflections.

**Length of curriculum:**

The curriculum is designed as three one-hour interactive workshops to be delivered over three to six months to allow for spaced skill practice in between sessions.

**Introduction:**

This mentorship training curriculum addresses critical gaps in mentorship quality and availability for emergency medicine faculty and learners. Faculty rarely are trained to be mentors, limiting mentoring quality and accessibility for mentees. We addressed these scarcity and quality gaps by offering concise experiential workshops for mid-career and senior faculty mentors to shift their mentoring mindset from expert advisor to curious and non-judgmental thinking partner, expanding their mentoring skills.

**Educational Goals:**

The aim of this curriculum is to train mentors to approach mentoring with a curious and non-judgmental mindset. The curriculum offers specific, practical exercises to increase mentors’ ability to foster a mentee-driven relationship that meets the needs of all mentees, which in turn enhances career development and faculty retention at the department level.

**Educational Methods:**

The educational strategies used in this curriculum include brief mentor check-ins, concise didactics with illustrative examples of personal experiences applying the workshop’s mentoring tools, peer mentoring practice time in paired breakout sessions, and large group debriefs.

**Research Methods:**

This curriculum was implemented at Oregon Health & Science University in three departments including Emergency Medicine, Ophthalmology, and the Hospitalist Division within Internal Medicine. This study was reviewed and approved by the Institutional Review Board at Oregon Health & Science University. We electronically collected survey data after each mentoring session to determine whether the participants found the workshops engaging and effective as well as whether their confidence and mentorship skills improved. The surveys gathered data using a 5-point Likert scale as well as free text responses.

**Results:**

Five faculty cohorts from three different departments completed the mentorship curriculum. Of the 35 total participants, 60%, 51%, and 5% completed the post-program feedback surveys for Workshops 1, 2, and 3, respectively. 95% of respondents rated the format as either very engaging or engaging and reported finding the workshop content very useful or moderately useful. All respondents stated they learned new information or skills they could apply to their own work or mentorship.

**Discussion:**

An accessible and applicable faculty development mentoring skills curriculum was implemented in three distinct academic departments with favorable assessment by participants. Combining concise lectures, personal mentorship stories, structured mentoring practice sessions, and group reflection effectively advanced faculty members’ skill and confidence in a mentee-centered mentorship approach. We learned that this curriculum is versatile enough to be tailored to various faculty settings and offers an engaging format that motivates faculty to voluntarily participate.

**Topics:**

Mentorship, communication skills, listening, faculty development, coaching, mentorship training.

## USER GUIDE

**Table:** 

List of Resources:	
Abstract	1
User Guide	3
Didactics and Hands on Curriculum Chart	9
Appendix A: Pre-work & Resources for Mentorship Workshops	12
Appendix B: Standard Agenda for Mentorship Workshops	14
Appendix C: Feedback Forms for Workshops	15
Appendix D: Mentorship Module 1	18
Appendix E: Mentorship Module 2	19
Appendix F: Mentorship Module 3	20

**Learner Audience:** This curriculum is designed for Faculty and Fellows.

**Length of Curriculum:** The curriculum is designed to offer three one-hour sessions with spaced practice in between according to faculty needs and time availability. The training could be completed over 3-6 months.

**Topics:** Mentorship, communication skills, listening, faculty development, coaching, mentorship training.

**Objectives:**

**Module 1 Overall Objective:** Faculty mentors will become proficient at using generous listening and structured career development exercises to support mentees in identifying their own career goals and decisions.

**Learning Objectives for Module 1:**

1. Faculty mentors will build awareness of positive past experiences in role as mentor and mentee.
2. Faculty mentors will consider advantages of practicing a generous and deliberate listening mindset as a mentor following workshop completion.
3. Faculty mentors will practice strategies for centering mentoring conversations on the mentee’s lived experiences following workshop completion.

**Module 2 Overall Objective:** Faculty mentors will become proficient at using structured values clarification exercises to support mentees in identifying their own career next steps.

**Learning Objectives for Module 2:**

1. Faculty mentors will assess their own values and career experience and apply that awareness during the practicum component of workshop.
2. Faculty mentors will practice appreciative inquiry around values of a mentee during practicum component of workshop.
3. Faculty mentors will prepare to apply values clarification as they support mentees’ in career/life planning strategy conversations following workshop completion.

**Module 3 Overall Objective:** Faculty mentors will become proficient at coaching mentees to say “no” to dys-opportunities.

**Learning Objectives for Module 3:**

1. Faculty mentors will describe criteria to distinguish opportunities from dys-opportunities following workshop completion.
2. Faculty mentors will practice coaching constructive ways to say no to opportunities that are not aligned with their mentee’s values and/or whose costs exceed benefits for mentees.
3. Faculty mentors will reflect on benefits and drawbacks of practicing a listening mindset with mentees during mentorship.

### Brief introduction

Mentorship is key to successful career development for emergency medicine faculty at academic health sciences institutions.^1^ Mentorship is often a scarce resource with surveys suggesting that fewer than half of academic physicians report having received mentorship during their careers.^2^,^3^ Some of the obstacles include a perceived lack of time and absence of organized mentorship programs. Other difficulties include mentor biases, the perception of mentors as agents of the institutional establishment, and mentors’ patronizing attitudes.^2^

Faculty mentors often approach mentorship as an opportunity to share wisdom gained from their career experience, assuming shared interests with their mentees. This style of mentorship can create mismatched expectations leading junior faculty to report less-than-adequate experiences from mentoring relationships.^2^,^4^ Our institution addressed these scarcity and quality gaps by offering a concise faculty development experience for mid-career and senior faculty mentors to shift their mentoring mindset from expert advisor to curious and non-judgmental listener, expanding the mentoring options and skills of existing faculty.

### Problem identification, general and targeted needs assessment

Mentorship evokes a mental model around expert/novice relationships where the mentor diagnoses career performance problems and gives useful advice. In medical school and residency, emergency medicine physicians learn and master patient conversations to elicit specific information and provide treatment guidance. These doctor-patient conversations and the classic advice-giving model of expert mentor guiding novice mentee differ significantly from the mentee-centered discussions required to mentor faculty from diverse backgrounds with unfamiliar interests or niches. Many mentors generalize from their own professional training and socialization to attempt to “help” junior colleagues by sharing their own experience. While sharing received wisdom may be valuable for some mentees when their values and circumstances align with their mentor, there is an unaddressed opportunity for mentors to practice a mentee-focused style of mentorship.

Coaching skills training offers a different model for supporting career development in others, typically using a structured process for goal setting and performance optimization. This curriculum incorporates a focus on curiosity and inquiry and a “coaching” style of collegial interaction without attempting to offer formal preparation as a coach. Our mentee-centered approach incorporates the mentees’ unique identities and their own lived experiences to help guide their careers as health professionals, scientists, and educators. By teaching the mentors active listening techniques, introspective exercises, and a more coach-like approach, faculty mentors gain new skills to mentor junior faculty.

Existing published curricula offer valuable resources for mentees, including tools to create a mentoring “mosaic”^5^ or effective mentoring “networks”^6^; others promote facilitated peer mentoring^7^ or group faculty development offerings for early career faculty.^8^ Few published curricula have focused on reframing dyadic mentoring from a traditional “expert” to a mentee-focused “coaching” style of mentoring. Recent notable exceptions include curricula focused on skills for mentoring across differences.^9^ Our curriculum contributes to the more limited body of resources supporting mentor listening skill development^10^,^11^ and strategies for restructuring the mentoring relationship.^12^

Our successful mentor skill-building intervention emphasizes the value of “humble inquiry”^13^ rather than “expert knowing” as a way of offering support for the career development of a colleague. Strong evidence exists for this inquiry-focused style that trains mentors to guide mentees in discovering their own unique priorities and strategies.^14^-^16^ Our brief workshops offer the chance for mentors to practice this approach with their peers within the workshop setting. Rather than telling mentees what they “should” do to be “successful” faculty, mentors learn to listen, draw out the priorities of the mentee, and support micro-actions toward greater career alignment and satisfaction.

Based on our initial needs assessment, this curriculum offers brief didactic illustrations of practical tools for mentee-centered mentoring conversations. We pair these teachings with interactive skill practice opportunities delivered in a small group, in-person or virtually, to maximize accessibility for busy faculty members. Each skill-building session uses real-time practice with peer mentors taking turns practicing (and experiencing) the skills in pairs, followed by reflection to notice the impact of this different style of mentoring. This “real-play” method was selected (as contrasted with role-play or scenario discussion) both to increase the relevance of the skill practice and to give each mentor a chance to experience being on the receiving end of each method. This publication details the key didactic teaching elements and the interactive skill practice setups for each of the three one-hour modules.

The target audience for this curriculum is mid-career to senior faculty seeking improved mentoring skills for an Emergency Medicine department mentorship program. Due to its positive feedback and successful implementation, the mentorship training was expanded to other departments including Ophthalmology, Hospitalist Medicine, and General Internal Medicine for their unique dyadic and group mentorship programs. This curriculum represents a novel contribution to the existing literature in its practical and unique approach to teaching skills for a mentee-centered approach to mentorship.

### Purpose of the curriculum

The global purpose of this curriculum is to address significant current limitations in quality and availability of mentoring relationships within academic health sciences. It aims to achieve this by:

- **Shifting mentor mindsets** from that of an expert advisor to a curious, non-judgmental listener and thinking partner.
- **Promoting mentee-driven relationships** that better support junior faculty, especially those with diverse backgrounds and unique lived experiences, allowing them to align their careers with their own values and goals.
- **Equipping mid-to-senior-level faculty** with practical communication and listening skills through brief, skill-building workshops to enhance their ability to mentor effectively across differences.

Ultimately, the intention is to create more supportive, productive, and satisfying mentoring experiences that meet the diverse needs of mentees in academic medicine.

### Objectives of the curriculum

**Module 1 Overall Objective:** Faculty mentors will become proficient at using generous listening and structured career development exercises to support mentees in identifying their own career goals and decisions.

### Learning Objectives for Module 1

1. Faculty mentors will build awareness of positive past experiences in role as mentor and mentee.
2. Faculty mentors will consider advantages of practicing a generous and deliberate listening mindset as a mentor following workshop completion.
3. Faculty mentors will practice strategies for centering mentoring conversations on the mentee’s lived experiences following workshop completion.

**Module 2 Overall Objective:** Faculty mentors will become proficient at using structured values clarification exercises to support mentees in identifying their own career next steps.

### Learning Objectives for Module 2

1. Faculty mentors will assess their own values and career experience and apply that awareness during the practicum component of workshop.
2. Faculty mentors will practice appreciative inquiry around values of a mentee during practicum component of workshop.
3. Faculty mentors will prepare to apply values clarification as they support mentees’ in career/life planning strategy conversations following workshop completion.

**Module 3 Overall Objective:** Faculty mentors will become proficient at coaching mentees to say “no” to dys-opportunities.

### Learning Objectives for Module 3

1. Faculty mentors will describe criteria to distinguish opportunities from dys-opportunities following workshop completion.
2. Faculty mentors will practice coaching constructive ways to say no to opportunities that are not aligned with their mentee’s values and/or whose costs exceed benefits for mentees.
3. Faculty mentors will reflect on benefits and drawbacks of practicing a listening mindset with mentees during mentorship.

### Educational Strategies

See Curriculum Chart.

Educational strategies used include optional pre- or post-workshop resources, brief lectures (highlighting evidence for specific strategies for mentoring junior faculty with different experiences), interactive skill practice sessions (via assigned paired breakouts), and large group discussions. Appendix A details the pre-work resources sent to participants prior to each workshop. The brief 15-minute lectures prior to the practice breakouts include personal stories and examples of experiences where the highlighted mentoring skill enables greater connection with junior faculty mentees. Group discussion among participants after peer mentoring skill practice breakouts supports reflection on impacts and the personal experience of both offering and receiving the highlighted skill in peer mentor pairs. Appendix B details the standard agenda and instructional pacing used for each workshop. At the end of each workshop, a feedback survey specific to each workshop was provided via the chat and/or emailed after the workshop, detailed in Appendix C.

### Results and tips for successful implementation

The Emergency Medicine department at Oregon Health & Science University implemented a small group mentoring program with junior mentees paired with mid-career to late-career faculty mentors with three trainings over nine months with 6–8 participants attending each session. Given that most academic faculty had no formal training on best practices as a mentor, we invited mentors to use methods for creating positive and productive relationships to support mentoring across differences without relying on unpredictable “chemistry” between people. Participation was voluntary and there were no prerequisites.

The curriculum is best implemented as a series of three one-hour workshops combining didactics illustrated with personal examples, skill-building practice breakouts, and large group discussions. The workshops can be delivered either virtually or in-person, depending on availability and departmental preference. The recommended workshop size is 6–8 faculty mentors.

Key strategies to successful implementation at our institution:

- Scheduling: Workshop schedules organized based on the availability of the participants to maximize participation in a virtual setting or an in-person setting outside of the workspace.
- Facilitators: One or two facilitators with prior experience leading interactive workshops, who clearly believe in and value mentee-centered mentorship.
- Didactics: Concise and evidence-based didactic sessions that incorporate facilitator’s personal stories to enhance impact and connection to the material.
- Practice: Peer mentoring practice in breakout pairs using provided prompts that teach how to implement the didactic training into actual mentorship conversations. Each participant was the mentor for half of the breakout time and the mentee for the other half of the session. We intentionally formatted these workshops to offer participants the chance to experience each skill, behavior, or tool from both the mentee and mentor role. Rather than using case studies and role play, we asked the participants to complete the exercises genuinely as a peer mentee bringing their own career experiences. With this formatting, the mentors could identify the value (both as a mentee and mentor) while practicing and growing these behaviors and skills for their mentorship roles.
- Reflection: Opportunity to reflect on the didactics and peer mentorship experience in the large group debrief including considering the perspectives of other participants.

The curriculum was implemented for five faculty cohorts in the departments of Emergency Medicine (8 participants), Ophthalmology (24 participants), and Hospital Medicine (8 participants) due to the success and positive feedback from the initial cohort. After each workshop, self-assessment surveys specific to each workshop were provided electronically after the workshop's completion via Qualtrics (see Table 1 for summary of responses). The survey response rates were highly variable but participation in all three workshops was robust within each departmental cohort, with no measurable dropoff in attendance.

For Workshop 1 (Deliberate Listening), 71% of respondents rated the format very engaging, 24% engaging, and 5% mildly engaging. Ninety-five percent reported finding the information very useful or moderately useful. All respondents said they learned new information or skills that could apply to their own work or mentorship. Many respondents stated they learned new listening skills such as “starting a question with ‘what’ rather than ‘why’” and asking questions about a mentee’s experiences rather than “relating to people with a lot of ‘me too’ responses.” The respondents commented that they appreciated the pre-work and content of the workshop. Several respondents who attended virtually suggested that future workshops be held in person.

For Workshop 2 (Values), all respondents rated it as very engaging (71%) or engaging (29%) and stated that the module offered useful information and insights. All participants stated they learned new information that could be applied to their own work or mentorship. The comments suggested they appreciated the workshop highlighting the importance of focusing on the mentee’s values (rather than assuming similar values to the mentor) and learning how to ask questions to better understand someone else’s values. Multiple participants said they learned from both skill practice and large group discussions.

For Workshop 3 (Opportunities/Dys-Opportunities), 100% of respondents rated it as very engaging and offered useful information and insights. Despite robust attendance at the third workshop, there were very few respondents to the third feedback survey. It is unclear why there were limited responses. Respondents stated that they learned new information from the summary of key recommended readings, including how to say “no” and offer alternatives; participants stated they found it a meaningful workshop with excellent delivery.

Overall, the positive reception and subsequent adoption by other departments within the institution provide strong encouraging evidence for the curriculum’s face validity and generalizability as a brief, effective, intervention for shifting mentoring mindsets to be mentee-centered.

### Evaluation and Feedback

From the data, the facilitators recognized the importance that participants placed on practicing mentoring techniques for true adoption. In response, didactics were condensed and the time spent in peer mentoring skills practice expanded. During the didactics, the facilitators adapted their personal stories to relate to the specific departmental audience. When the participants empathized with the examples given during the didactics sessions, the material was more relatable, memorable, and demonstrates practical applicability. It was also clear that emphasizing “real-play” over “role-play” in peer mentoring practice was highly effective. It allowed the participants to personally experience the impact of the skills from both sides, increasing relevance and buy-in as well as building connections between the mentors.

### Associated Content

Appendix A: Pre-work & Resources for Mentorship Workshops.docx

Appendix B: Standard Agenda for Mentorship Workshops.docx

Appendix C: Feedback Forms for Workshops.docx

Appendix D: Mentorship Module 1 Slides

Appendix E: Mentorship Module 2 Slides

Appendix F: Mentorship Module 3 Slides

## Supplementary Information







## Figures and Tables

**Table:** 

Topic	Recommended Educational Strategy	Educational Content	Objectives	Learners	Timing, Resources Needed	Recommended Assessment, Milestones Addressed
Module 1: Generous/Deliberate Listening	Interactive reflection time on current state of mentoring relationships. Lecture sharing personal mentorship stories and rationale for generous and deliberate listening as a fundamental mentorship skill (Appendix D). Structured peer mentoring skill practice in paired breakout. Large group debrief and reflection on insights and takeaways.	Rationale for mentors to be a thinking partner with their mentees rather than an expert problem solver. Practice skill of generous and deliberate listening emphasizing curiosity, vulnerability, and seeking understanding. Introduce potential impacts of unconscious biases in mentoring relationships. Optional resources (videos, articles, reflection exercises) for participant review before or after workshop.	Build awareness of positive experiences in role as mentor and mentee in past. Consider advantages of practicing a generous and deliberate listening mindset as a mentor. Practice strategies for counteracting unconscious biases.	Mid-career and senior faculty serving as mentors	10 minutes (introductions, reflection on current mentoring relationship) 15 minutes (didactics with personal story examples) 25 minutes (paired peer mentor skill practice) 10 minutes (large group reflection on skill practice) Instructors: 1 plus videoconference “producer” to set up and manage breakout groups in virtual format Equipment: PowerPoint and screen for in-person, videoconference software in virtual format Post-session assessment (Appendix C)	Self-assessment
Module 2: Values/Goals	Interactive reflection on current mentoring relationship. Lecture with framework for Career/Life Planning Strategy through values clarification exercises (Appendix E). Offer alternative model of “integration” of work, self, family and community rather than work/life “balance.”	Engage in values assessment on their own values and career experience. Practice inquiry around values of a mentee. Apply values clarification in context of career/life planning strategy. Reinforce value of listening mindset emphasizing curiosity, vulnerability, and seeking understanding.	Describe personal values including work, community, self, and family elements. Practice inquiry around values of a mentee. Reflect on experience practicing listening mindset with mentees since prior workshop.	Mid-career and senior faculty serving as mentors	10 minutes (reflection on current mentoring relationship, application of skills from prior workshop) 15 minutes (didactics with personal story examples) 25 minutes (paired peer mentor skill practice) 10 minutes (large group reflection) Post-session assessment (Appendix C) Instructors: 1 plus videoconference “producer” to set up and manage breakout groups in virtual format Equipment: PowerPoint and screen for in-person, videoconference software in virtual format	Self-assessment
Module 3: Opportunities /Dys-Opportunities	Interactive reflection time on current state of mentoring relationships. Lecture sharing personal examples of dys-opportunities and ways to say no constructively (Appendix F). Structured peer mentoring skill practice in paired breakout. Large group debrief and reflection on insights and takeaways.	Framework to distinguish opportunities from dys-opportunities. Assess costs and benefits of a new opportunity. Strategize how to say no in a constructive way. Reinforce value of listening mindset emphasizing curiosity, vulnerability, and seeking understanding.	Describe criteria to distinguish opportunities from dys-opportunities. Prepare constructive ways to say no. Reflect on experience practicing listening mindset with mentees since prior workshop.	Mid-career and senior faculty serving as mentors	10 minutes (reflection on current mentoring relationship, application of skills from prior workshop) 15 minutes (didactics with personal story examples) 25 minutes (paired peer mentor skill practice) 10 minutes (large group reflection) Post-session assessment (Appendix C) Instructors: 1 plus videoconference “producer” to set up and manage breakout groups in virtual format Equipment: PowerPoint and screen for in-person, videoconference software in virtual format	Self-assessment

**Table 1:** Survey Responses by Workshop Topic

	Generous/Deliberate Listening (n = 21/40 participants, 53% response rate)	Values/Goals (n = 18/40 participants, 45% response rate)	Opportunities/Dys-Opportunities (n = 2/40 participant, 5% response rate)
*The module achieved its objectives in an engaging format.*			
Very Engaging	15 (71%)	12 (71%)	2 (100%)
Engaging	5 (24%)	5 (29%)	0 (0%)
Neither Engaging nor Not Engaging	1(5%)	0 (0%)	0 (0%)
*The module offered useful information and insights.*			
Very Useful	13 (62%)	10 (59%)	2 (100%)
Moderately Useful	7 (33%)	7 (41%)	0 (0%)
Minimally Useful	1(5%)	0 (0%)	0 (0%)
*Did you learn new information that you can apply to your own work or mentorship?*			
Yes	21 (100%)	18 (100%)	2 (100%)
*Please rate your confidence based on a five-point scale (1=low and 5=high).*			
	“I understand the practice of generous listening.”	“I can identify my core values as they apply to my career goals.”	“I understand the different frameworks to determine an opportunity from a dys-opportunity.”
5	10 (53%)	9 (53%)	0 (0%)
4	8 (42%)	6 (35%)	2 (100%)
3	1(5%) 2	(12%) 0	(0%)
2-1	0 (0%)	0 (0%)	0 (0%)
*Please rate your confidence based on a five-point scale (1=low and 5=high).*			
	“I will be able to practice generous listening skills with my mentee.”	“I am able to engage with my mentee to help them identify professional and personal goals that represent their values.”	“I am able to discuss frameworks to determine an opportunity from a dys-opportunity with my mentee.
5	8 (38%)	3 (18%)	1 (50%)
4	11 (52%)	12 (71%)	1 (50%)
3	2 (10%)	2 (11%)	0 (0%)
2-1	0 (0%)	0 (0%)	0 (0%)
*Please rate your confidence based on a five-point scale (1=low and 5=high).*			
	“I will be able to start a conversation with my mentee about setting expectations.”	“I have access to tools to begin mentoring in a structured manner.”	“I am able to guide my mentee on how to say ‘no’ to a dys-opportunity.”
5	9 (43%)	5 (29%)	0 (0%)
4	10 (48%)	10 (59%)	1 (50%)
3	2 (10%)	1 (6%)	1 (50%)
2	0 (0%)	1 (6%)	0 (0%)
1	0 (0%)	0 (0%)	0 (0%)
*Please rate your confidence based on a five-point scale (1=low and 5=high).*			
	[no additional question]	“I can initiate conversation with my mentee about how to strategize to accomplish their goals.”	[no additional question]
5		6 (35%)	
4		9 (53%)	
3		2 (12%)	
2		0 (0%)	
1		0 (0%)	

**Figure:**
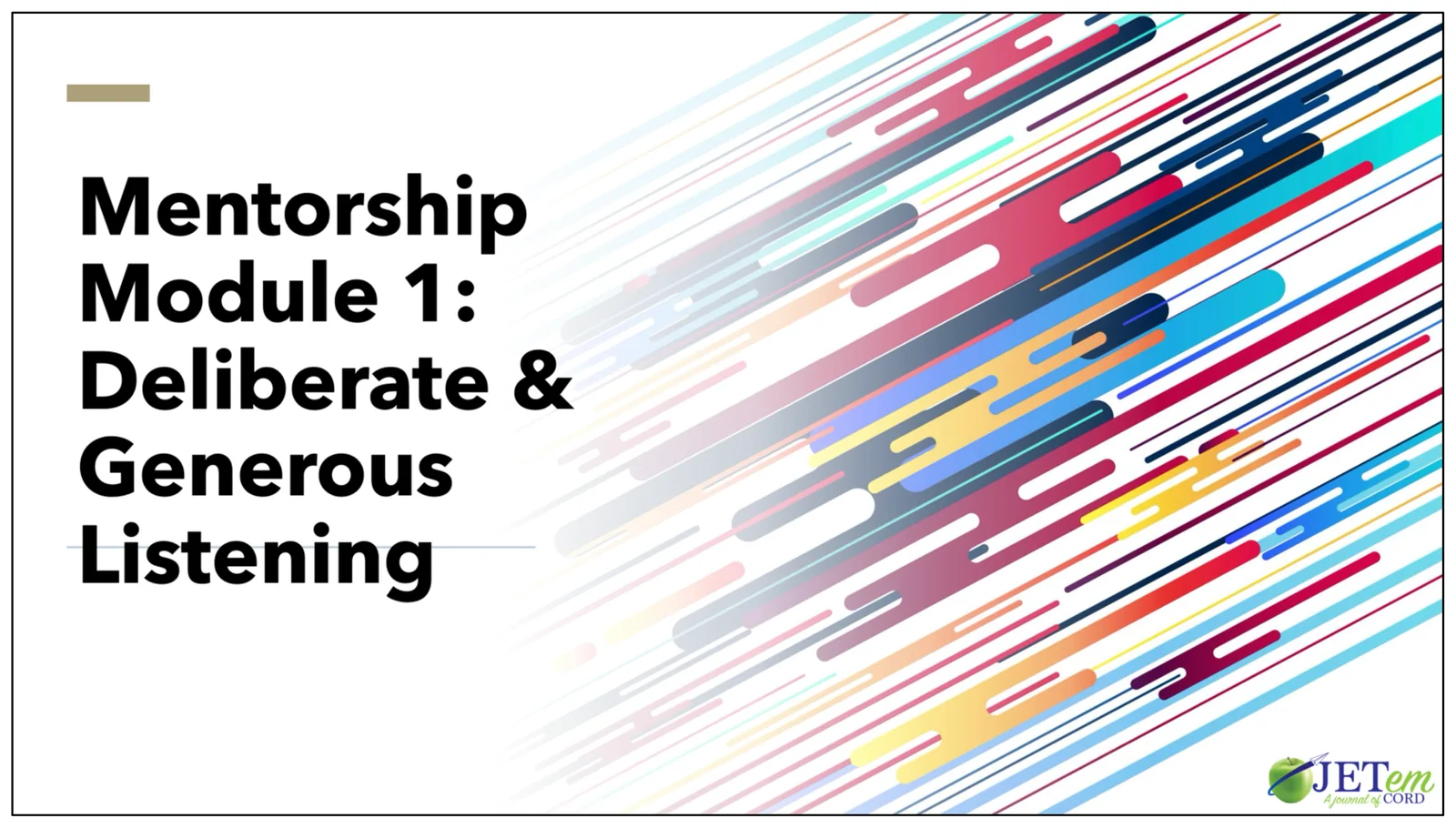
Please see associated PowerPoint

**Figure:**
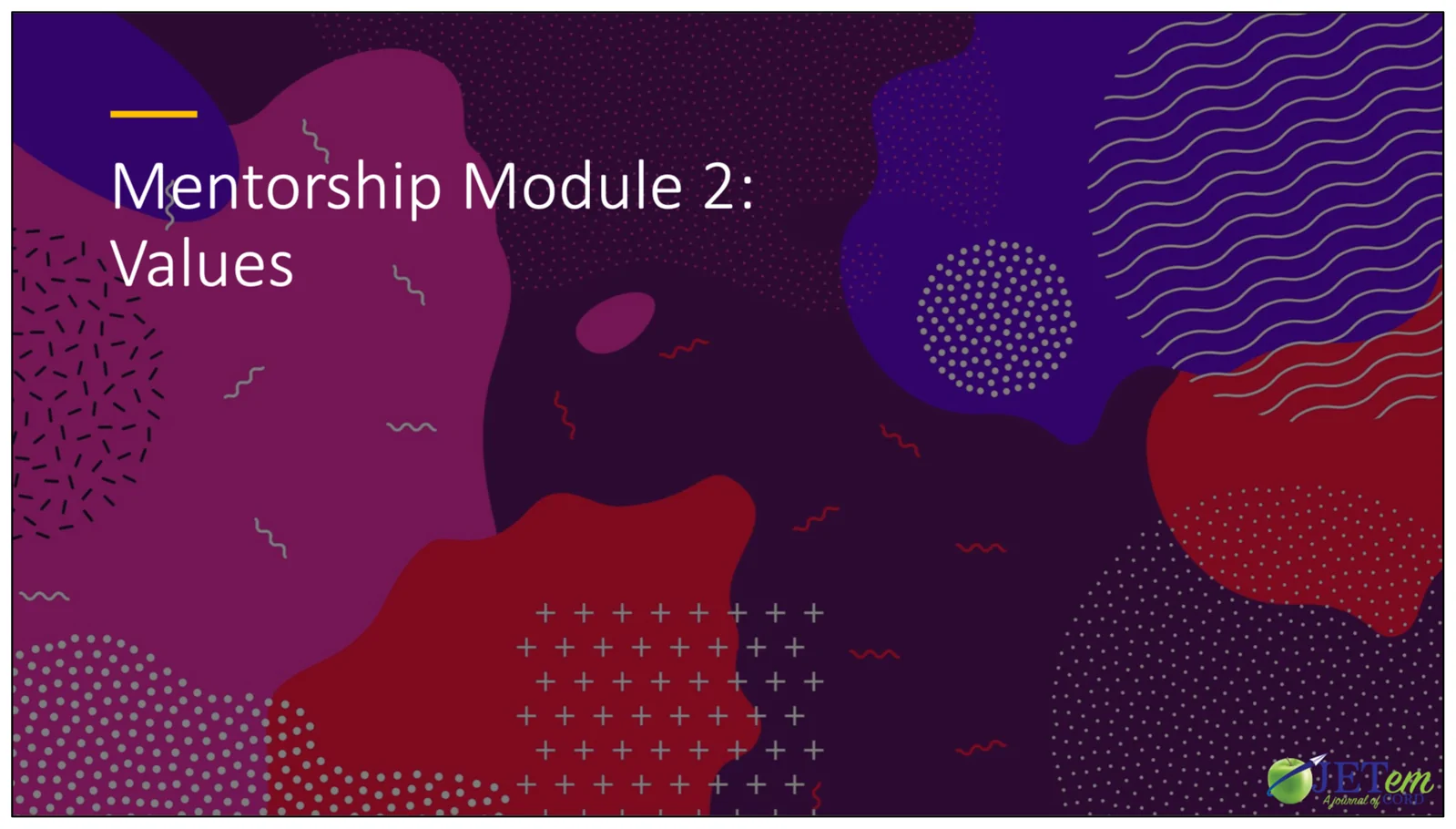
Please see associated PowerPoint

**Figure:**
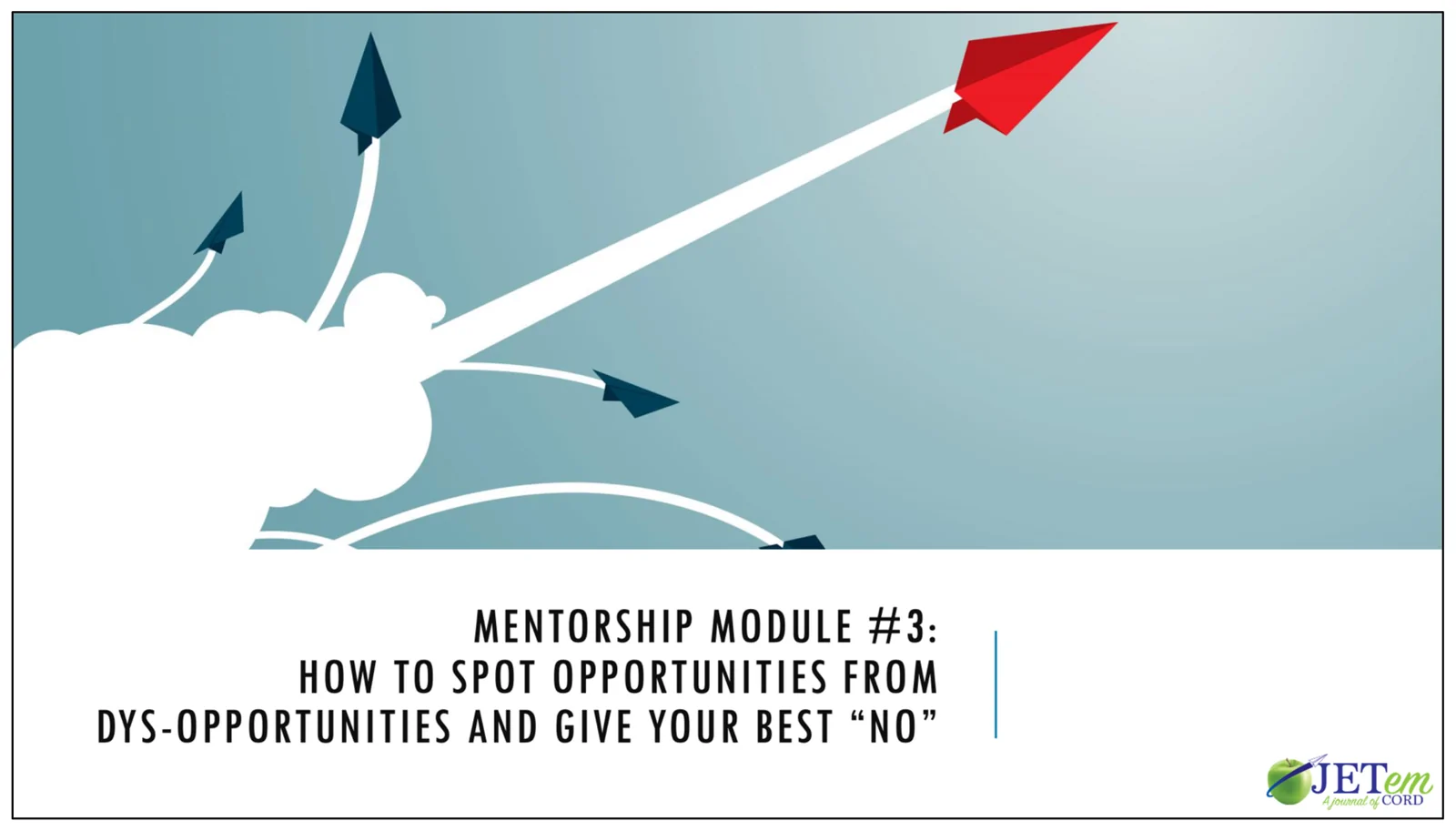
Please see associated PowerPoint

## Appendix A Pre-work & Resources for Mentorship Workshops

### Workshop 1: Deliberate and Generous Listening

*Resources about listening:*

- Ury W. The power of listening. TEDxSanDiego. Accessed March 13, 2025. https://www.youtube.com/watch?v=saXfavo1OQo
- Polaneczky R. The power of deliberate listening. TEDxPhiladelphia. Accessed March 13, 2025. https://www.youtube.com/watch?v=A343tlP5iUA
- Stanier, MB. How to tame your advice monster. TEDxUniversityofNevada. Accessed March 13, 2025. https://www.youtube.com/watch?v=Kl0rmx7aa0w

*Resources about acknowledging personal/unconscious bias including gender, race, age, class, specialty, academic interest:*

- Banaji, MR. Welcome to outsmarting implicit bias. Accessed March 13, 2025. https://youtu.be/-9OjvkR7Ynk
- Banaji, MR. The universe inside your mind. Accessed March 13, 2025. https://www.youtube.com/watch?v=n-hX0StkQdY&t=3s
  ○ Take one or more Implicit Association Tests: Accessed March 13, 2025. https://implicit.harvard.edu/implicit/takeatest.html https://implicit.harvard.edu/implicit/iatdetails.html
- Bumsted T. Generational Differences Trigger Tapes Videos
  ○ Generational bias: Accessed March 13, 2025. http://www.youtube.com/watch?v=C504i9ysGsY
  ○ (Start watching at 4:25, Trigger Tape 3)
  ○ Mentoring across generational divide: Accessed March 13, 2025. https://www.youtube.com/watch?v=8EFbAXIsVa0
  ○ Work-life balance video: Accessed March 13, 2025. http://www.youtube.com/watch?v=HNwaQRp6Qoo

### Workshop 2: Values-Based Mentoring

*Resources about values assessment:*

- Online values sort: Accessed March 13, 2025. http://www.pztools.org/valuesort/-faq
- Brown B. Dare to lead list of values. https://brenebrown.com/resources/dare-to-leadlist-of-values/
- Friedman, SD. *Total Leadership*. Boston, MA: Harvard Business Review Press; 2014.
- Burnett B, Evans D. *Designing Your Life: How to Build a Well-Lived, Joyful Life.* New York, NY: Alfred A. Knopf; 2016.

### Workshop 3: Opportunities vs. Dys-opportunities

*Resources about decision making and saying no:*

- Brooks, A. Overwhelmed? Just say ’NO.’ The science of how to stop saying yes to everything – and be happier. *The Atlantic Monthly*.2024;February 29.
- Gittell JH. *High Performance Healthcare: Using the Power of Relationships to Achieve Quality, Efficiency, and Resilience.* McGraw-Hill Education; 2018.
- Goleman D, Boyatzis RE, McKee A. *Primal Leadership: Unleashing the Power of Emotional Intelligence.* Boston, MA: Harvard Business Press; 2013.
- Kegan R, Lahey, LL. *How the Way We Talk Can Change the Way We Work: Seven Languages for Transformation.* Hoboken, NJ: Jossey-Bass, 2002.
- McKeown G. *Essentialism: The Disciplined Pursuit of Less.* Crown Business, 2014.
- Patterson K, Grenny J, McMillan R, Switzler A. *Crucial Conversations: Tools for Talking When Stakes Are High*. Maidenhead, England: McGraw-Hill Contemporary; 2002.
- Reeder J. *Black Holes and Energy Pirates: How to Recognize and Release Them.* Crossing Press; 2001.
- Scott S. *Fierce Conversations: Achieving Success at Work & in Life One Conversation at a Time.* New York, NY: Penguin Books; 2002.
- Stone D, Patton B, Heen S. *Difficult Conversations: How to Discuss What Matters Most.* 10th anniversary ed. New York: Penguin Books; 2010.
- Stone D, Heen S. *Thanks for the Feedback: The Science and Art of Receiving Feedback Well (Even When It Is Off-Base, Unfair, Poorly Delivered, and Frankly, You’re Not in the Mood).* New York, NY: Viking; 2014.
- Straus SE, Sackett DL, editors. *Mentorship in Academic Medicine*. Chichester, UK: Wiley- Blackwell; 2013.
- Tan CM. *Search Inside Yourself: The Unexpected Path to Achieving Success, Happiness (and World Peace).* New York, NY: HarperOne; 2012.
- Ury W. *The Power of a Positive No: How to Say No and Still Get to Yes.* New York, NY: Bantam Books; 2007.

## Appendix B Standard Agenda for Mentorship Workshops (60 minute total)

- Opening Introduction and Participant Engagement (10 minutes)
  ○ Introductions/Greetings
  ○ Reflection on Current Mentoring Relationship
    ▪ Headline about positives or struggles within the relationship
- Didactics (15 mins)
  ○ Lecture Examples in Appendices D, E, and F
  ○ Personal stories/reflections on topics incorporated into lectures
- Paired Peer Mentoring Exercises (25 minutes)
  ○ Mentorship Trainings each have their questions/discussion topics included in the didactic component (Appendices D-F)
    ▪ 10–12 minutes – person #1 in pair designated as the mentee and person #2 as the mentor
    ▪ 10–12 minutes – person #2 designated as the mentee and person #1 as the mentor
- Large Group Reflection (10 minutes)
  ○ Discuss experience of mentoring and being the mentee in a generous listening setting
  ○ Discuss the relevance of the topics discussed for mentorship

## Appendix C Feedback Forms for Workshops

### Mentorship Training Workshop #1 Feedback Form

1. The module achieved its objectives in an engaging format.
  **Table:**
  Strongly Agree	Somewhat Agree	Neutral	Somewhat Disagree	Strongly Disagree
2. The module offered useful information and insights.
  **Table:**
  Strongly Agree	Somewhat Agree	Neutral	Somewhat Disagree	Strongly Disagree
3. Did you learn new information and strategies that you can apply to your own work or mentorship?
  Yes. If yes, please describe:
  No
4. Please assess your confidence in the following based on a five-point scale
  - I understand the practices of generous listening.
    **Table:**
    5 High	4	3	2	1 Low
  - I am able to practice generous listening skills with my mentee.
    **Table:**
    5 High	4	3	2	1 Low
  - I can articulate my image of success in academic medicine.
    **Table:**
    5 High	4	3	2	1 Low
5. Please tell us what we should do differently next time to enhance learning. (Please comment on any/all of the following: Pre-work, Activities, Timing/pacing of module, Logistics of training, Discussions.)
  - What should we add?
  - What should we remove?
  - What should we keep the same?

### Mentorship Training Workshop #2 Feedback Form

1. The module achieved its objectives in an engaging format.
  **Table:**
  Strongly Agree	Somewhat Agree	Neutral	Somewhat Disagree	Strongly Disagree
2. The module offered useful information and insights.
  **Table:**
  Strongly Agree	Somewhat Agree	Neutral	Somewhat Disagree	Strongly Disagree
3. Did you learn new information and strategies that you can apply to your own work or mentorship?
  Yes. If yes, please describe:
  No
4. Please assess your confidence in the following based on a five-point scale
  - I can identify my core values as they apply to my career goals.
    **Table:**
    5 High	4	3	2	1 Low
  - I am able to engage with my mentee to identify their values.
    **Table:**
    5 High	4	3	2	1 Low
  - I can discuss with my mentee how their current time, projects, and goals align with their values.
    **Table:**
    5 High	4	3	2	1 Low
  - I am able to incorporate self-reflective exercises for my mentee into my mentoring conversations.
    **Table:**
    5 High	4	3	2	1 Low
  - I can initiate conversation with my mentee about their values and goal setting.
    **Table:**
    5 High	4	3	2	1 Low
5. Please tell us what we should do differently next time to enhance learning. (Please comment on any/all of the following: Pre-work, Activities, Timing/pacing of module, Discussions.)
  - What should we add?
  - What should we remove?
  - What should we keep the same?

### Mentorship Training Workshop #3 Feedback Form

1. The module achieved its objectives in an engaging format.
  **Table:**
  Strongly Agree	Somewhat Agree	Neutral	Somewhat Disagree	Strongly Disagree
2. The module offered useful information and insights.
  **Table:**
  Strongly Agree	Somewhat Agree	Neutral	Somewhat Disagree	Strongly Disagree
3. Did you learn new information and strategies that you can apply to your own work or mentorship?
  Yes. If yes, please describe:
  No
4. Please assess your confidence in the following based on a five point scale
  - I understand the different frameworks to determine whether something is an opportunity or a dys-opportunity.
    **Table:**
    5 High	4	3	2	1 Low
  - I can discuss applying frameworks to determine an opportunity from a dysopportunity with my mentee.
    **Table:**
    5 High	4	3	2	1 Low
  - I can guide my mentee on how to say “no” to a dys-opportunity.
    **Table:**
    5 High	4	3	2	1 Low
5. Please tell us what we should do differently next time to enhance learning. (Please comment on any/all of the following: Pre-work, Activities, Timing/pacing of module, Logistics of training, Discussions.)
  - What should we add?
  - What should we remove?
  - What should we keep the same?
